# Effect of Surface Treatment on Stiffness and Damping Behavior of Metal-Metal and Composite-Metal Adhesive Joints

**DOI:** 10.3390/polym15020435

**Published:** 2023-01-13

**Authors:** Adeela Nasreen, Muhammad Kashif Bangash, Khubab Shaker, Yasir Nawab

**Affiliations:** National Center for Composite Materials, School of Engineering and Technology, National Textile University, Faisalabad 37610, Pakistan

**Keywords:** surface treatment, adhesion, thermal properties, composite-metal joints

## Abstract

In aerospace and automotive applications, composite materials are used as a major structural material along with metals. Composite-metal and metal-metal joining are very crucial in such structures. Adhesive bonding is commonly used for this purpose. Since such structures are exposed to varying temperatures and dynamic loads, it is essential to investigate the response of such joints under thermomechanical loading. Though various studies have been reported in the literature to assess the thermomechanical properties of composites, adhesives, and their joints, the effect of the surface treatment of metals and composites on the improvement in the thermomechanical behavior of the joints has not been reported. The metal and composite surfaces were modified using chemical etching techniques. The interaction between adhesives and adherends was studied using the DTMA technique in compression mode. Anodizing treatment on aluminum alloys improved the stiffness properties of metallic joints to 36% and decreased the damping to 23%, while chemical treatment on composite and metal adherends increased the stiffness of composite-metal joints to 34% and reduced the energy dissipation to 20%.

## 1. Introduction

Aerospace materials must be lighter in weight and structurally efficient, besides having high stiffness and thermal stability. Therefore, a combination of metals and composites is used in aerospace structures. Due to the differences between the two, the joining of these components has been a serious problem [1,2].

Adhesive bonding is the most efficient and durable method for assembling composite parts into metal parts [3,4,5]. To achieve a durable bond at an elevated temperature, efficient surface preparation for joining adherents must ensure the following features: contaminant removal, high surface energy, etc. Numerous surface pretreatments such as sandblasting, chemical etching, and anodizing are used for this purpose, as cited by Nasreen et al. [6,7]. After surface modification of composites and metals, their joints are developed with adhesives [8], their bonding performance could be assessed by the lap shear test, and their viscoelastic behavior with temperatures, time, and frequency could be determined using thermomechanical analysis [9,10].

The viscoelastic behavior of adhesives and adhesive joints is characterized by the elastic modulus, viscous modulus, and loss factor determined by thermomechanical analysis (TMA/DMA). In thermomechanical analysis, the storage modulus appears at the initial temperature and indicates the elastic response of materials due to strong crosslinking between polymeric chains. While the loss modulus appears at the middle temperature, where the molecular mobility starts and represents the viscous response of a material. The balance between the elastic and viscous responses is represented by the tan delta (loss factor) [11].

**Figure 1:**
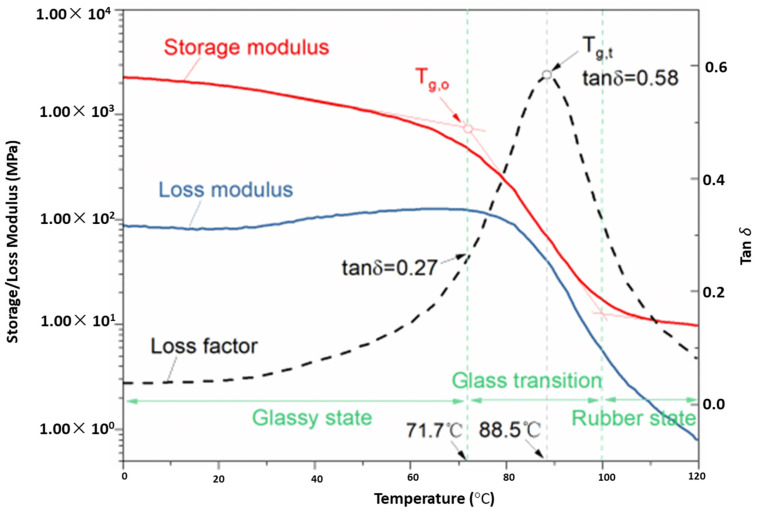
Dynamic mechanical thermal analysis results of film adhesive [9].

Figure 1 represents the thermomechanical results of a film-like epoxy as a function of time and temperature. The outcomes demonstrate the mechanical strength of the epoxy, exhibiting three clear regions. Region 1 is known as the brittle state, through which the elastic modulus declined slightly with the variation in temperature due to the stretching of secondary bonds. Region 2 is known as the phase transition, during which the elastic modulus descends strongly due to the breaking of secondary bonds. Region 3 is also known as the rubbery state, in which the elastic modulus remains lowermost and stable [12,13,14].

A comparison of temperature resistance and lap shear strength of adhesives has been shown in Table 1. It was assessed from the lap shear strengths of different adhesives that AF 163-2 adhesive has the highest lap shear strength and LY 564 has the least lap shear strength, so using similar adherends and similar surface preparations on both adherends with different adhesives will change the bonding performance of the joint. Moreover, it was observed from the temperature resistance of adhesives that Aremco 2310 has a temperature resistance from −55 °C to 165 °C, while LY 564 has a temperature resistance from 25 °C to 150 °C. Therefore, both will perform best in different temperature ranges. Ultimately, adhesive joints developed using these adhesives will have different temperature stabilities [15,16,17,18].

In the past, Ke et al. [9] studied the effect of temperature on adhesive bond strength with a dynamic mechanical analyzer (DMA). Amuthakkannan et al. [15] evaluated the viscoelastic properties of carbon fiber-reinforced plastics using DMA. Sperandio et al. [10] and Krogh et al. [11] studied the mechanical and thermal properties of PU adhesives using DMA. Causse et al. [16] studied the mechanical behavior of epoxy adhesives in bonded aluminum assemblies using DMA. Previous literature focused on the thermomechanical properties of adhesives [19], composites [15,20], and wooden joints [21] and the numerical investigations of adhesive joints [13]. The treatment of adherends before joining them with Aremco 2310 has not been reported in the literature to date. The metal/metal and metal/composite adhesive joints are subjected to dynamic thermomechanical analysis. 

In a previous paper published in a polymer composite journal, various surface pretreatments were applied to metal and composite adherends, and the effect of chemical surface treatment on the performance of adhesive joints was characterized under static and impact loadings [14]. However, in the present study, the chemical surface pretreatments on metal and composite substrates were applied, and their effect on the stiffness and damping behaviors of adhesive joints was studied using dynamic thermomechanical analysis (DTMA).

## 2. Materials

The aluminum alloy (al 7075-T6) was 0.6 mm thick and had a density of 2.6 g/cm^3^; it was used as a metal substrate and was sourced by Shandong Nuozhong Steel Co., Ltd, China. The sourced aluminum alloy is being used in the aerospace industry due to its high strength and low ductility. The glass fabric was procured from HD FIBERGLASS, Xingtai, China. The carbon fiber tow (T800, 12K) with 12,000 filaments was imported from Hexcel, a U.S. company.

LY 564/Ardur 22962, epoxy resin was supplied by Huntsman International, USA. LY 564 epoxy is a Bisphenol A-Epichlorohydrin epoxy with cycloaliphatic polyamine as its hardener. The epoxy resin has a low viscosity, a density of 1.1–1.2 g/cm^3^, a glass transition temperature of 80 °C, and failure elongation between 3.5 and 8%. The weight ratio of epoxy resin to hardener was 4:1, and the curing cycle of epoxy resin was 80 °C for 1 h and 150 °C for 2 h. The Aremco 2310 adhesive is paste-like, black in color, has a density of 1.35 g/cm^3^, and has a lap shear strength of 33 MPa at room temperature. The Aremco 2310 adhesive was selected due to its ultra-high bond strength at room temperature [14]. The features of composites, metals, and adhesives are provided in Table 2.

**Figure 2:**
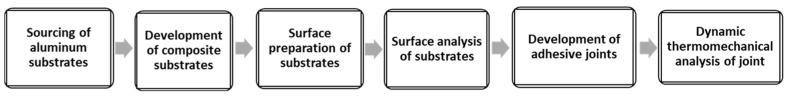
Manufacturing process of study.

The carbon fiber/epoxy prepreg was produced using a solvent dip procedure. Then 20 plies of carbo/epoxy prepreg were laid in a [0,90]_s_ symmetric configuration. After that, 2 plies of woven glass/epoxy were arranged on top of the carbon/epoxy prepreg. The whole setup was cured in a press machine at 150 °C for two hours under a 0.1-ton load. The fiber volume fraction of the composite was 65 ± 1%. The glass-carbon hybrid composite was developed to avoid galvanic corrosion problems in metal-composite adhesive joints. the manufacturing process of current research work is given in Figure 2.

### Surface Preparation

**Figure 3:**
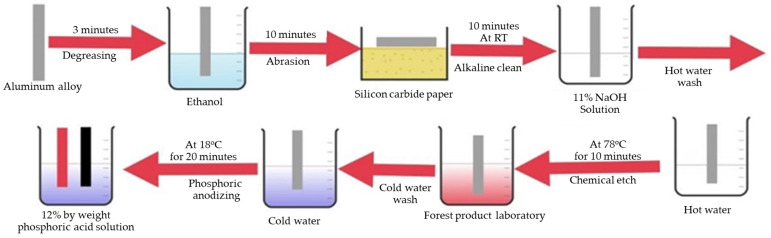
Schematic diagram for an anodizing process for aluminum alloy.

The surface of the metals was prepared using a series of treatments mentioned in Figure 3. The aluminum alloy (Al 7075-T6) was degreased with ethanol for 5 min. Then the mechanical treatment was performed using silicon-carbon sandpapers of 100 grits for 15 min. Following that, alkaline cleaning was performed at 40 °C for 10 min with an 11% NaOH solution to wipe off silicon and magnesium hydroxide from the metal substrate. After hot water washing of the metal surface for 1 min, chemical etching was performed using mixtures of sulfuric acid, water, and sodium dichromate. After cold washing for one-minute, phosphoric acid anodizing was performed to add a fresh oxide layer to the metal surface. At last, the primer was applied using 1% glycidoxypropyltrimethoxysilane solution for 10 min. The silane primer was cured on an anodized surface at 90 °C for 15 min. The whole treatment process of the metal alloy was performed as per standard ASTM D 3933.

**Figure 4:**
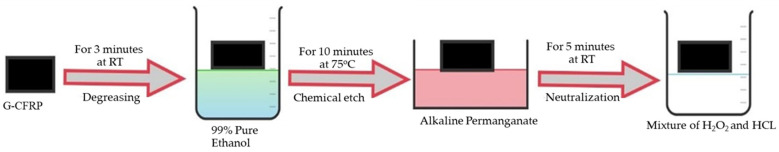
Schematic of chemical etching process on composites.

To prepare the composite substrate surface, two techniques were used: Peel ply treatment and chemical etching. The peel ply is a fabric layer that is affixed to the laminate during the compression stage of curing. There are three steps involved in chemically etching a composite surface such as degreasing, oxidation, and neutralization. To get rid of the dust, the composite surface was degreased in an ethanol solution for three minutes. Following that, potassium permanganate (70 g/L) and sodium hydroxide (60 g/L) solutions were used to create an alkaline permanganate etchant. After that, distilled water was used to dilute the alkaline permanganate solution. The alkaline permanganate etching procedure took place at 75 °C for 10 min. The etching process attacks polymer-solvent and polymer-polymer bonds and splits the crosslinking sites. Following that, the neutralization process of the composite surface was performed using a mixture of hydrogen peroxide (2%) and sulfuric acid (2%). The neutralization process eliminates the oxide coating built up on the composite surface because of etching. At last, rinsing of the composite surface was performed with water, as suggested by Kirmann [22] and Young [23] (Figure 4).

## 3. Adhesive Joint Fabrication

The adhesive joint was produced by sandwiching a thin layer of adhesive between the composite substrate and metal substrates. The whole setup was then left for curing at room temperature for one day followed by post-curing at 150 °C in the oven for 2 h. The thickness of the adhesive layer was maintained at 0.6 mm using constant loading. The dimensions of composite/metal adhesive joints were 7 mm × 7 mm × 3.2 mm in length, width, and thickness direction, respectively. Three replicates of each joint were produced as mentioned in Table 3.

**Figure 5:**
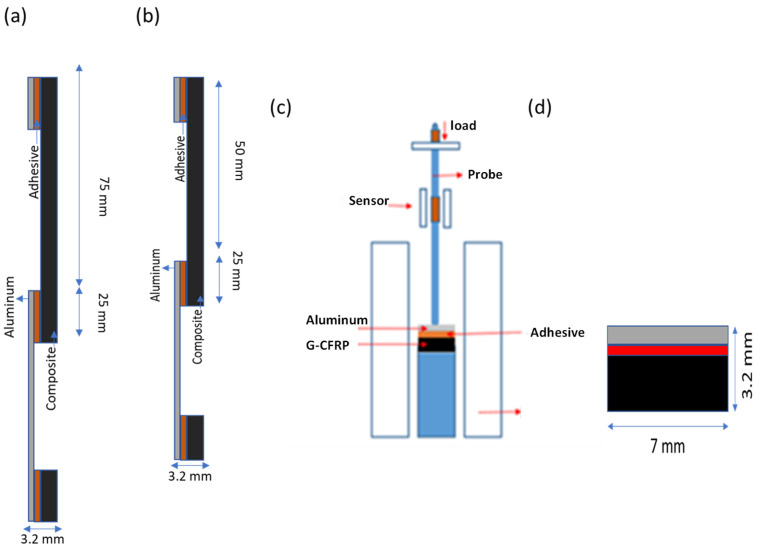
(**a**) Single lap shear strength test sample, (**b**) Izod impact strength test sample, (**c**) DTMA test sample, and (**d**) setup for DTMA test.

The metal and composite substrates after surface modification were examined using an optical microscope and a contact angle goniometer. The substrates were then bonded, and a single lap shear strength test was performed at a crosshead speed of 1 mm/min as per standard ASTM D 1002. The sample dimensions were 25 mm × 175 mm × 3.2 mm in width, length, and thickness directions. Izod Impact strength testing was performed as per standard ISO 180. The dimensions of the impact sample were 10 mm × 125 mm × 3.2 mm in width, length, and thickness directions (Figure 5). Both tensile and impact strength testing were performed at room temperature and at a relative humidity of 65 %. Thermomechanical analysis of adhesive joints was performed using a compression probe in the dynamic mode on the TMA Q400 equipment. The test was conducted in the temperature range of 30 °C to 150 °C at a ramp rate of 3 °C/min, dynamic force of 0.1 N, and a frequency of 0.5 Hz. The resultant sinusoidal strain and phase difference (δ) are noted. Using these outputs, the elastic modulus (E’), viscous modulus (E”), and loss factor (tan delta) are calculated as a function of time, stress, or temperature [21]. 

## 4. Discussion

### Surface Texture Analysis

**Figure 6:**
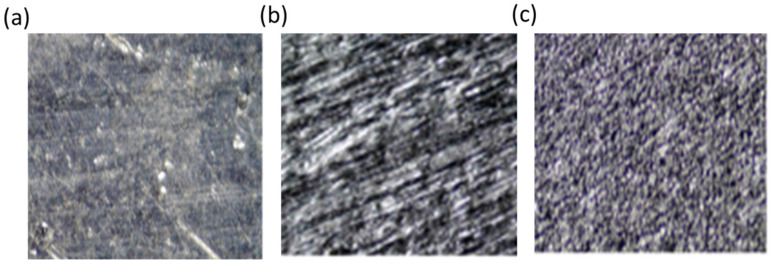
Comparison of surface textures of metals at 45X: (**a**) untreated, (**b**) abraded, and (**c**) abraded and anodized.

Figure 6 compares the morphologies of untreated, abraded, and anodized aluminum surfaces. The untreated surface is covered with scratches, oil, and dust particles, as shown in Figure 6a. After degreasing and abrasion, an unstable oxide layer was removed, and a macroroughness was created, as shown in Figure 6b. After chemical etching and the anodizing treatment, a fresh, porous oxide layer (bubble like surface) was developed (Figure 6c), the surface area was increased, and the adhesion strength between the metal and adhesive was improved, as suggested by Digby et al. [24].

**Figure 7:**
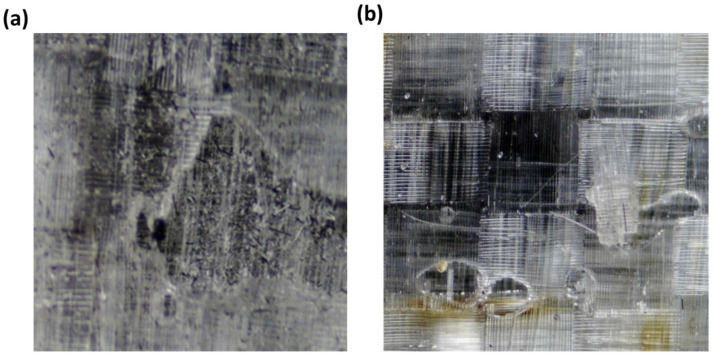
Comparison of the surface texture of composite substrates at a magnification of 35X: (**a**) peel ply-treated and (**b**) peel ply followed by chemical etching.

Figure 7 compares the surface texture of the composite surface after the peel ply treatment and the chemical etching methods. It was observed that the peel-ply fabric interacts with matrix materials, etches its surface macroscopically, and increases the surface area of the composite. However, some residue of the peel ply is left on the surface after treatment, which needs to be removed for better adhesion purposes [25,26].

When the chemical etching process was achieved after peel ply removal, it removed the contaminants of the peel ply, cleaned the composite substrate, and added micro-roughness to the composite substrate surface (Figure 7b). The additional process after peel ply treatment etched the surface homogeneously at the microlevel (without disturbing bulk properties) [18]. This allowed for an improvement in the contact area and interlocking points between the adhesive and composite substrate surfaces. Thus, the adhesion strength between the composite and adhesive was enhanced.

## 5. Wettability Performance Test

**Figure 8:**
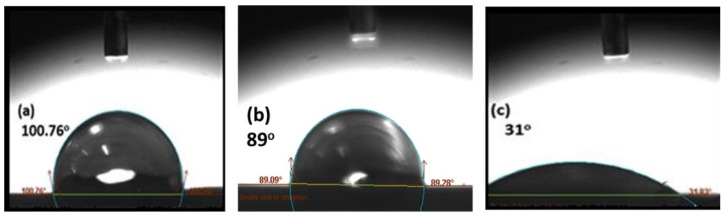
Comparison of wettability performance test results of metal substrates at a magnification of 45X (**a**) untreated, (**b**) abraded, (**c**) and abraded plus anodized.

The mean contact angles of the metal surfaces after the surface modification are presented in Figure 8. The measurements showed that the contact angle of distilled water on degreased and abraded metal surfaces decreased to 89° compared to untreated surfaces. The degreasing and mechanical treatment removed oil pollutants and dust particles from the metal surface; macroroughness was observed, and hydrophilicity increased. After etching with a phosphoric acid solution, the distilled water contact angle on a metal surface decreased to 31°. The lowest contact angle of water on an anodized surface provided the highest hydrophilicity and greatest wettability performance of joints. The improvement in the wettability of the anodized surface was primarily due to the creation of a thin, porous aluminum oxide coating.

**Figure 9:**
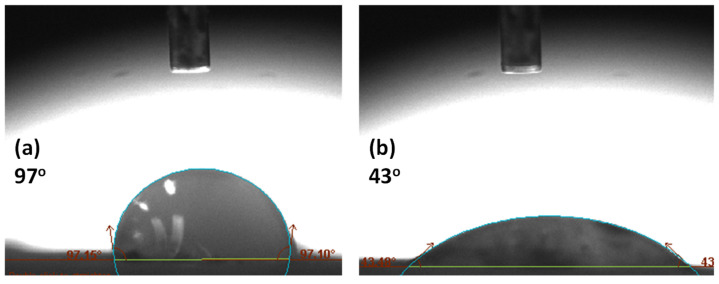
Comparison of contact angle results of the composite substrate at a magnification of 45X: (**a**) peel-ply treated and (**b**) peel ply removal followed by chemical etching.

Figure 9 indicates the wettability results of the composite substrate after the surface pretreatment. The peel ply treatment on a composite surface added macroroughness but was hydrophobic, as shown in Figure 9a. Although, when the composite surface was chemically etched after peel ply removal, the contact angle was reduced to 43°. Therefore, the hydrophilicity increased due to the presence of microroughness, and the adhesion strength was improved.

## 6. Effect of Surface Treatment on Mechanical Performance of Joints

The ASTM D 1002 standard was used to measure the lap shear strength of adhesive joints. During testing, the gauge length and crosshead speed were kept at 150 mm and 1 mm/min, respectively. The Izod impact strength test was performed according to ISO 180 [14]. Table 4 summarizes the lap shear tensile [27] and impact strength of metal-metal and composite-metal joints. It was observed that metal/metal bonded joints (Mech-mech), in which both metal adherends were treated mechanically, keep lower lap shear strength and lower impact strength than metal/metal anodized joints. The mechanical treatment on a metal substrate imparted macroroughness and mechanical interlocks between the metal surface and the adhesive layer. The mech-mech-bonded joints failed due to mix-mode fracture (cohesive in the adhesive and adhesive at the composite-metal interface). The mechanical treatment of a metal substrate improved the adhesive bonding between the metal and adhesive, but the adhesion strength was found to be insufficient for achieving chemical bonding between the adhesive and adherends, while, the metal-metal anodized bonded joints, in which metal substrates were mechanically treated and anodized, had the highest lap shear strength. The anodizing treatment on an abraded surface created a porous layer on a metal surface. Therefore, both the abraded surface and the porous layer produced both mechanical interlocking and chemical bonding between the metal and adhesive. The joint failed due to the cohesive failure of the metal substrate because the joint strength exceeded the metal adherends’ strength.

Composite-metal adhesive joints (PP-mech, Chem-mech), in which the metal substrate was mechanically treated, and the composite surface was peel plied or chemically etched, possessed lower lap shear strength and lower Izod impact strength than others. The failure of these joints took place due to an adhesive failure at the metal-composite interface. The adhesive has displayed weak bonding with the metal surface and effective bonding with the polymer composite. Composite-metal adhesive joints (PP-ano, Chem-ano), in which the metal substrate was mechanically treated and anodized while the composite surface was peel-ply treated or chemically etched, possessed higher lap shear strength and greater impact strength than others. Due to the series of treatments on metal alloys changed surface roughness, wettability performance of adherends. When adhesive joints were produced, they induced mechanical interlocking and chemical bonding between adhesives and adherends. Thus, bonding performance is improved. The failure of these joints occurred due to the delamination between GFRP and CFRP plies. The anodizing treatment created a porous layer over the abraded surface and improved the joint strength to a greater level, as cited by Nasreen et al. [14].

## 7. Effect of Surface Treatment on Joint Stiffness

**Figure 10:**
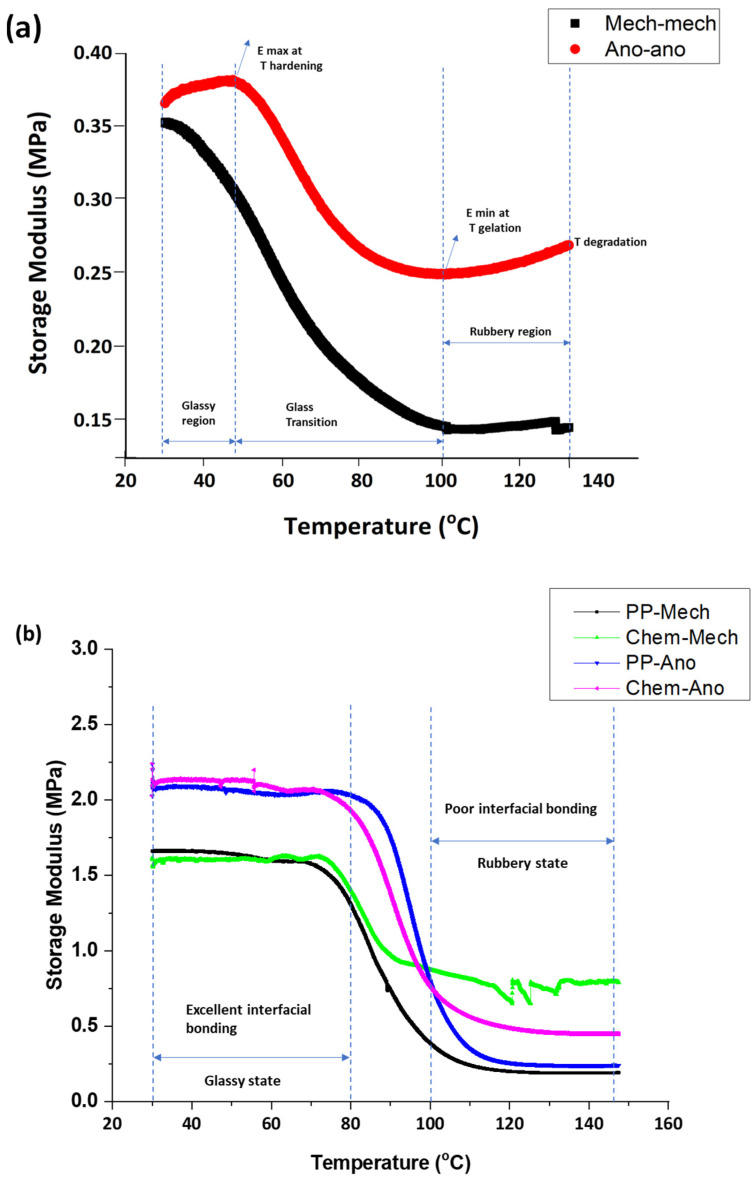
Comparison of storage modulus of bonded joints: (**a**) metal-metal joint and (**b**) composite-metal joint.

Figure 10 relates the stiffness of joints with the temperature of metallic joints. The results show that the stiffness of metal-metal joints was found to be highest at the hardening temperature (35 °C) because the polymeric chains of the adhesive are tightly packed, the crosslinking between the adhesive and composites [28] is the largest, and the thermomechanical properties of the joints are highest. At the glass transition temperature (58 °C), the elastic modulus of joints declines sharply because the secondary bonds of the adhesive break, and the stiffness of joints also decreases. The relative elastic modulus (difference between the maximum and minimum modulus) of joints from 58 °C to 98 °C was found to be lower than that from 35 °C to 58 °C because of the decrease in mechanical strength of the adhesive with temperature change. The storage modulus of the adhesive joints was lowest and most stable within the 98 °C to 150 °C temperature range. In this region, the polymeric chains of the adhesive start flowing, and no more interaction exists to transfer stress. The adhesive joints have the lowest stiffness at elevated temperatures.

Mech-mech joints, in which both metal substrates were degreased and abraded, kept showing lower stability with temperature than ano-ano joints. Moreover, mech-mech joints store less elastic energy than ano-ano joints. The mechanical treatment on metal surfaces added macroroughness to the surface and increased hydrophilicity but was insufficient for achieving high stiffness in joints. Whereas ano-ano joints, where both metal adherends were mechanically treated and anodized, possessed higher stiffness and higher thermal stability than mech-mech joints. The anodizing treatment added a microporous aluminum oxide layer between the metal and the adhesive, thus, responsible for improving the stiffness of joints at all temperature ranges (Figure 10a).

Figure 10b compares the behavior of the stiffness of joints with a temperature ramp for composite-metal-bonded joints with different surface treatments. The relative stiffness of all composite/metal joints was found to be greatest from 30 °C to 72 °C because the crosslinking between the adhesive and adherents is the largest and the thermomechanical properties of the joints are the highest. As the glass transition phase started (72 °C), the stretching and breaking of secondary bonds started, and the relative elastic modulus of the joints declined sharply until 100 °C (gelation temperature), therefore, the stiffness of the joints started to decrease, and the mechanical strength of the adhesive joints decreased. From 100 to 150 °C (gelation to degradation temperature), the relative elastic modulus of the joints was found to be the lowest, and interactions between joining components were lost, so the transfer of stress between the adhesive and joining adherends was the lowest.

The PP-ano and Chem-ano joints in Figure 10b, in which the composite substrates were treated (chemically etched or peel-ply treated) and the metal surface was abraded and anodized, possessed higher stiffness and storage modulus until the glass transition temperature than the PP-mech and Chem-mech joints. To improve the stiffness of the joints, a combination of mechanical and chemical surface treatments was used. This resulted in mechanical interlocking and chemical bonding between adhesives and joining components.

Compared to the PP-ano and PP-mech joints, the Chem-ano and chem-mech joints in Figure 10b, which involved chemically etching the composite substrates and treating the metal surface (abrading or anodizing), are more stable at high temperatures. This may be attributed to the elimination of the impurities, and a uniformly etched composite surface after chemical etching. As a result, the adhesive and composite could be retained micromechanically, increasing the composite’s stiffness and stability at high temperatures.

## 8. Effect of Surface Treatment on Damping Behavior of Joints

### 8.1. Loss Modulus

**Figure 11:**
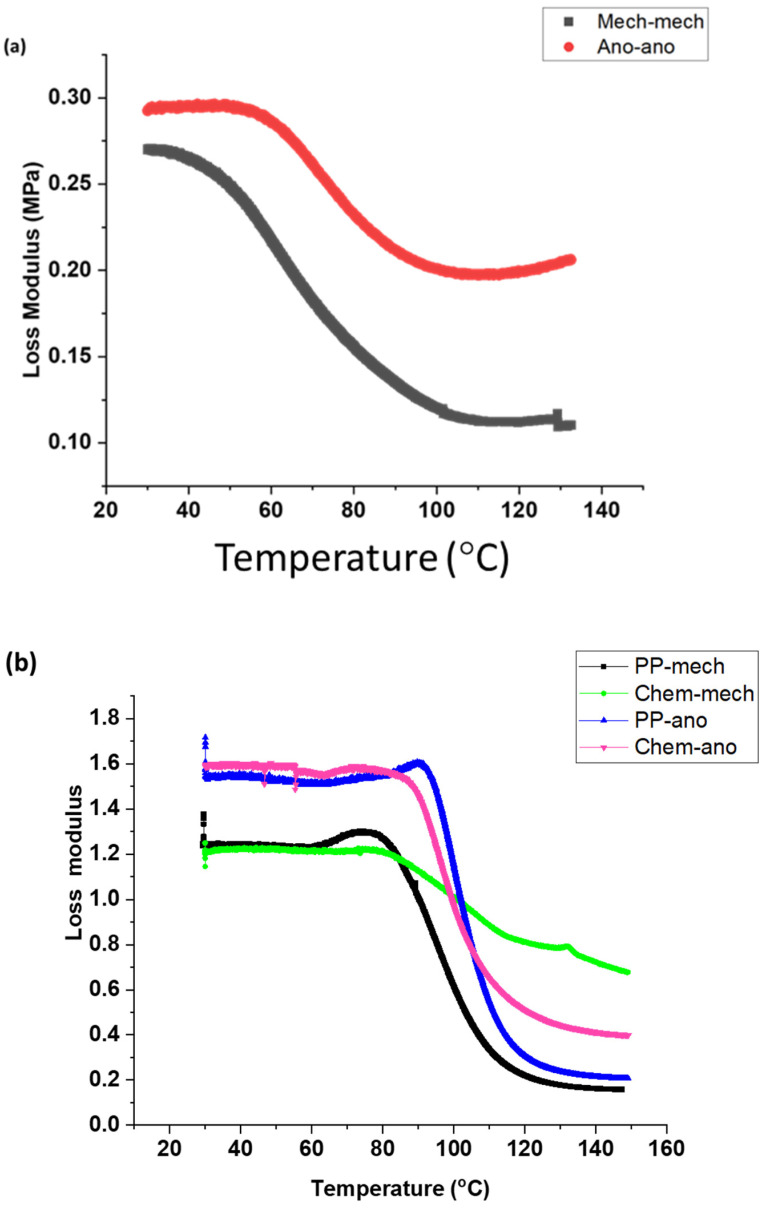
Loss modulus versus temperature: (**a**) metal-metal joints and (**b**) composite-metal joints.

Figure 11a indicates the viscous response of metallic adhesive joints to temperature changes. The loss modulus of metallic joints declines with the increase in temperature because the resistance to deformation of joints decreases with the increase in temperature. The higher viscous modulus of metallic joints up to 67 °C shows that higher frictional slides occur in a glassy region that gives higher energy dissipation. At 67 °C, a glass transition occurs, resistance to the flow of polymeric chains decreases, and energy dissipation reduces. Now the joints are less stiff and rubberier. From 100 °C to 150 °C, the polymeric chains of joints are flowing, thus causing the viscous modulus and energy dissipation to be lowest.

Figure 11b correlates the loss modulus of composite-metal adhesive joints with surface treatments on adherends. The higher loss modulus of composite-metal joints at 80 °C indicates that higher resistance to the flow of polymeric chains occurs in the glassy region. At 80 °C, the molecular mobility started, bond breakage dominated, and the interfacial bonding between polymeric chains reduced, therefore, the loss modulus of composite-metal joints declined dominantly. From 100 °C to 150 °C, the resistance to deformation is at a minimum, the energy dispersion is lowest, and the loss modulus is minimal.

Chem-ano joints, which include chemically treating the composite substrate and anodizing the metal surface, suffer larger losses than chem-mech joints. This behavior is observed because the anodizing treatment on a metal surface created a porous layer between the adhesive and the metal and possessed the largest resistance to deformation and the highest energy dispersion.

The Chem-ano and chem-mech joints have stronger resistance to deformation than the PP-mech and PP-ano joints at high temperatures because chemical etching of the composite produces micromechanical interlocking and has a high thermal stability at elevated temperatures.

### 8.2. Tan Delta

**Figure 12:**
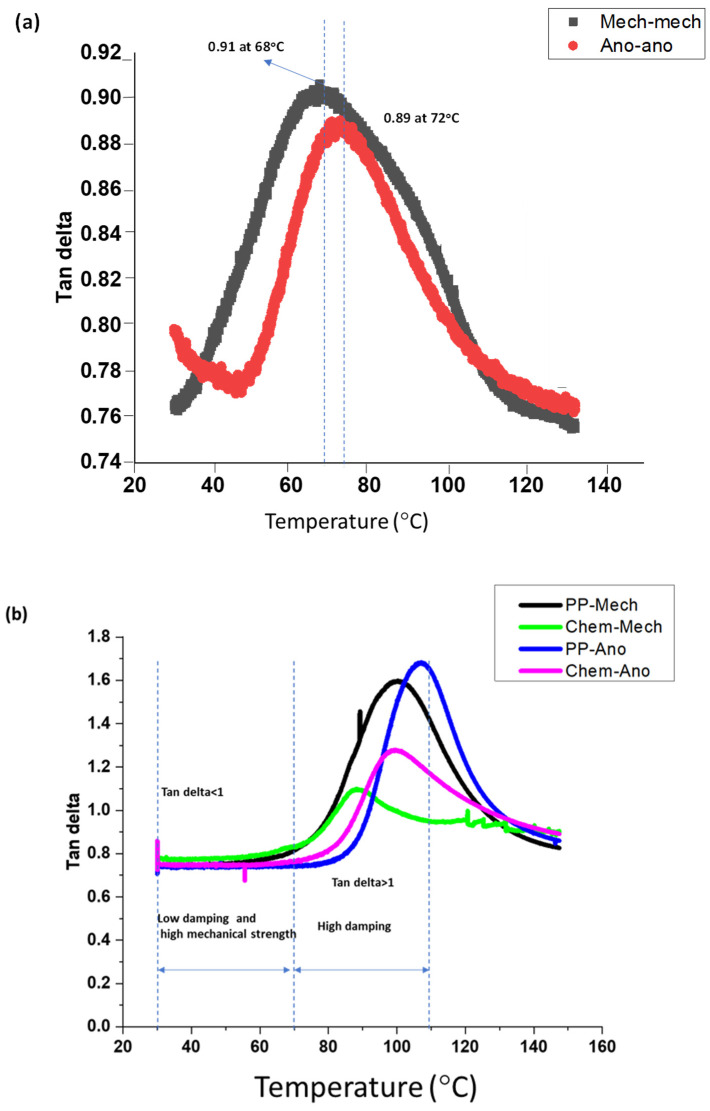
Comparison of a damping factor of bonded joints as a function of temperature: (**a**) metal-metal joints and (**b**) composite-metal joints.

Figure 12a compares the tan delta (loss factor) of metallic bonded joints with the temperature ramp after surface modification. With the temperature rising, the tan delta increases in the glassy region, reaching a high value at a phase transition point, and then beginning to decline in the rubbery phase. The tan delta of composite/metal joint curves greater than one signifies poor interfacial bonding between adhesive and adherends, and the peak point of the loss factor (tan delta) curves indicates the glass transition temperature of the adhesive joints, after which the mechanical strength of the joint degrades sharply. The mechanically treated and anodized metal substrates used in ano-ano bonded joints have greater glass transition temperatures than the mech-mech joints. The tan delta of the ano-ano joint is 0.89 at 70 °C, which is less than the tan delta of the mech-mech joint. This is because the ano-ano joints showed low damping against dynamic forces.

Figure 12b compares the tan delta (loss factor) versus the temperature of the composite/metal-bonded joints after surface modification. The outcome shows that the loss factor of all bonded specimens was less than 1 from 30 °C to 72 °C due to the strong interfacial bonding and low energy dissipation between the adhesive and adherends. At 72 °C, the tan delta is increasing with temperature and achieves a tan delta greater than 1 at the glass transition and then begins to decrease in the rubbery region [10].

The PP-mech and Chem-mech bonded joints had a larger loss factor (1.2 to 1.4) at the glass transition temperature than the PP-ano and Chem-ano joints because the composite substrate surface was peel-ply treated or chemically etched and the metal was abraded. To obtain excellent interfacial bonding, metal alloys require additional surface treatments. 

The PP-Ano and Chem-ano joints, on the other hand, had a smaller loss factor (1 to 1.2) at the glass transition temperature because they treated the composite substrate (using peel-ply treatment or chemical etching) and mechanically prepared and anodized the metal surface. The adhesion strength was improved, and the damping was decreased to 28% on metal surfaces after mechanical and anodizing treatments.

## 9. Conclusions

The experimental results demonstrate an alternative route for measuring the viscoelastic properties of adhesive joints at elevated temperatures. Through surface treatment of metals and composites, a weak boundary layer is removed, surface roughness is added, and hydrophilicity is increased. This allowed for improving the stiffness and storage modulus and reducing the damping properties of adhesive joints at low temperatures. All adhesive joints (metal/metal, composite/metal) show good performance until the glass transition temperature, while after the glass transition temperature, their mechanical strength starts decreasing and becomes lowest at the highest temperature.

A combination of mechanical and anodizing treatments on metal adherends improved the stiffness properties of metallic joints to 36% and reduced the damping to 23%, while the combination of chemical etching and anodizing treatments on composite and metal adherends improved the composite/metal joint stiffness to 34% and reduced the damping to 20%. The combination of mechanical and anodizing treatments is recommended for metallic joints, while a combination of chemical etching and anodizing is preferred for composite/metal joints in the aerospace industry, where stiffness is required.

## Figures and Tables

**Table 1:** Comparison of adhesives’ properties.

Brand Name	Physical Appearance	Lap Shear Strength	Temperature Resistance
Aremco 2310	Paste	33 MPa	−55 °C–165 °C
LY 564	Film	9 MPa	25 °C–150 °C
Redux 312	Film	27 MPa	25 °C–100 °C
Epibond 100	Paste	22.8 MPa	25 °C–150 °C
AF163-2	Film	39 MPa	−55 °C–121 °C

**Table 2:** Specification of materials [21].

Technical Name	Thickness (mm)	Tensile Strength in Longitudinal Direction (MPa)	Tensile Modulus Longitudinal Direction (GPa)
G-CFRP	2	415	57
Al 7075-T6	0.6	575	71
Aremco 2310	0.6	33	-

**Table 3:** Experimental design of bonded joints.

Serial No.	Type of Joint	Bonded Samples Coding	Surface Treatment	Actual Samples
1	Metal-metal	Mech-mech	Mechanical treatment on both metal substrates	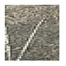
2	Metal-metal	Ano-ano	Mechanical and anodizing treatment on both metal substrates	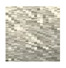
3	Composite-metal	PP-mech	Peel ply treatment on composite and mechanical treatment on metal	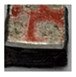
4	Composite-metal	PP-ano	Peel ply treatment on composite and anodizing on metal	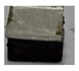
5	Composite-metal	Chem-mech	Peel ply and chemical etching on composite and mechanical treatment on metal	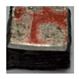
6	Composite-metal	Chem-ano	Peel ply and chemical etching on composite and anodizing on metal	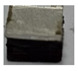

**Table 4:** Mechanical performance of adhesive joints.

Type of Joint	Type of Surface Treatment	Tensile Strength (MPa)	Impact Strength (KJ/m^2^)	Failure Surfaces after Tensile Test	Failure Surfaces after Impact Test
Mech-mech	Mechanical	11.5	119	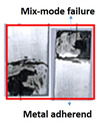	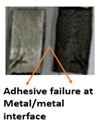
Ano-ano	Anodizing	12.6	224	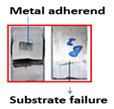	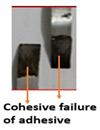
Mech-pp	Mechanical-peel ply	8.41	50	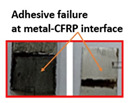	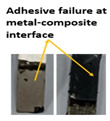
Ano-pp	Anodizing-peel ply	9.83	95	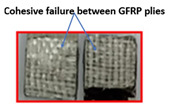	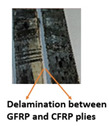
Mech-chem	Mechanical-chemical	11.7	102	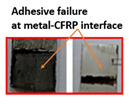	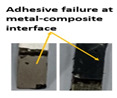
Ano-chem	Anodizing-chemical	12.5	147	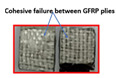	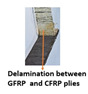

## Data Availability

The data will be made available as per demand.
